# Critical Period of Weed Control in Aerobic Rice

**DOI:** 10.1100/2012/603043

**Published:** 2012-06-18

**Authors:** M. P. Anwar, A. S. Juraimi, B. Samedani, A. Puteh, A. Man

**Affiliations:** ^1^Institute of Tropical Agriculture, Universiti Putra Malaysia, Serdang 43400, Selangor, Malaysia; ^2^Department of Agronomy, Bangladesh Agricultural University, Mymensingh 2202, Bangladesh; ^3^Department of Crop Science, Universiti Putra Malaysia, Serdang 43400, Selangor, Malaysia; ^4^Rice and Industrial Crops Research Centre, Malaysian Agricultural Research and Development Institute, Kuala Lumpur 50774, Malaysia

## Abstract

Critical period of weed control is the foundation of integrated weed management and, hence, can be considered the first step to design weed control strategy. To determine critical period of weed control of aerobic rice, field trials were conducted during 2010/2011 at Universiti Putra Malaysia. A quantitative series of treatments comprising two components, (a) increasing duration of weed interference and (b) increasing length of weed-free period, were imposed. Critical period was determined through Logistic and Gompertz equations. Critical period varied between seasons; in main season, it started earlier and lasted longer, as compared to off-season. The onset of the critical period was found relatively stable between seasons, while the end was more variable. Critical period was determined as 7–49 days after seeding in off-season and 7–53 days in main season to achieve 95% of weed-free yield, and 23–40 days in off-season and 21–43 days in main season to achieve 90% of weed-free yield. Since 5% yield loss level is not practical from economic view point, a 10% yield loss may be considered excellent from economic view point. Therefore, aerobic rice should be kept weed-free during 21–43 days for better yield and higher economic return.

## 1. Introduction

Critical period of weed control (CPWC) is an integral part of integrated weed management (IWM) and can be considered the first step to design weed control strategy [1]. The CPWC is the period of crop life cycle during which weeds must be controlled to prevent unacceptable or economic yield loss [2–4]. In theory, presence of weeds before or after CPWC will not pose a threat and should not cause significant yield loss [3, 5]. Thus, crop yield obtained by weeding during CPWC is almost similar to that obtained by the full season weed-free conditions. In general, one-third of the crop life cycle is considered as critical for weed control. A long critical period is the indication of less competitive crop or more competitive weeds and vice versa [6]. Studying CPWC could help identify residual action required for preemergence herbicide, improve timing and reduce the amount of postemergence herbicide applications [2], and thus may lessen potential environmental and ecological degradation [7]. Therefore, CPWC has been the subject of extensive research in field crops for the last few decades [8, 9].

Critical period of weed control has got a beginning and an end as well. The beginning of CPWC is determined by estimating critical time for weed removal (CTWR) after which weed control must be initiated to ensure potential yield [7, 10]. The end of CPWC, on the other hand, is determined by estimating critical weed-free period (CWFP) required from planting to avoid irrevocable yield loss [3, 4]. The CPWC is determined by calculating the time interval between CTWR and CWFP. Critical period of weed control has commonly been reported as day after seeding (DAS), but due to differences in planting dates and environment, this may generate results with more variability among locations, seasons, and cultivars. Therefore, CPWC determination based on DAS has been criticized by many researchers [7]. In recent studies, CPWC has been reported as growing degree days (GDDs) because it is a biologically meaningful measure of time required for plant growth and development [11], and therefore, it would be applicable for comparing critical period across different agroclimatic conditions [4].

The CPWC is likely to be unique for every crop species because of their morphophysiological makeup [3], but it is not an inherent property of the crop rather a measurement of crop-weed-environment interaction [12]. Whether or not each crop has got CPWC is arguable [13]. Some crops have CPWC, while others may not. Since the introduction of CPWC concept in 1960s, countless studies have been conducted around the globe to determine the CPWC in rice [14–19]. In West Africa, Johnson et al. [15] estimated CPWC for lowland irrigated rice as 0–32 DAS in wet season, while 4–83 DAS in dry season to obtain 95% yield. In Malaysia, based on the 5% yield loss, Begum et al. [17] concluded that flood-irrigated rice must be kept weed-free from 14 to 28 DAS, while Juraimi et al. [18] suggested that direct-seeded rice should be kept weed-free for 2–71 DAS in saturated condition and 15–73 DAS in flooded condition. In the Philippines, Chauhan and Johnson [19] estimated CPWC of rice as between 18 and 52 DAS to obtain 95% of weed-free yield.

Aerobic rice, growing rice in nonpuddled and nonsaturated soil like an upland crop, is gaining popularity day by day as a waterwise technology [20, 21]. But this technology is impeded by high weed pressure because of dry tillage and aerobic soil conditions [22, 23], and hence, weed management has been a challenge for this promising technology. The question remains unanswered is the following: will the aerobic rice system alter the CPWC? It is not unlikely that a switch from flood-irrigated to aerobic system will certainly bring a change in species composition, pressure, and emergence pattern of weeds resulting in a new dimension of weed-crop competition usually not existent in flood-irrigated rice system. Studies on single and multispecies weed interference effect on rice have been extensively conducted in different rice ecosystems, but a very little effort has been made so far to determine CPWC in aerobic rice system. Extrapolation of the findings obtained from flood-irrigated rice system to determine the CPWC in newly introduced aerobic rice system seems unwise which warrants urgent studies to determine CPWC in aerobic rice for its sustainability. The purpose of this study was to define and estimate the critical period of weed control for direct-seeded aerobic rice towards developing a less-herbicide-dependent weed management strategy. Other significant purpose was to evaluate the effect of different weed interference period on some agronomic and physiological traits of rice under aerobic soil conditions.

## 2. Materials and Methods

### 2.1. Experimental Site and Soil

 The trial was carried out during off-season 2010 (May–July) and main season 2010/2011 (November–January) under field condition at the experimental farm, Universiti Putra Malaysia, Malaysia (3°00′ 21.34′′ N, 101°42′ 15.06′′ E, 37 m elevation). The local climate was hot humid tropic with high humidity and abundant rainfall. During the off-season, monthly average maximum and minimum temperature and relative humidity ranged from 33.5 to 35.0°C, 23.6 to 24.6°C, and 93.3 to 93.9%, respectively, while rainfall, evaporation, and sunshine hours ranged from 4.2 to 10.8 mm/day, 3.65 to 4.90 mm/day, and 5.90 to 7.37 hrs/day, respectively. During the main season, monthly average maximum and minimum temperature and relative humidity ranged from 31.7 to 33.3°C, 22.9 to 23.8°C, and 94.1 to 94.6%, respectively, while rainfall, evaporation, and sunshine hours ranged from 6.1 to 9.9 mm/day, 2.64 to 4.66 mm/day, and 3.95 to 6.34 hrs/day, respectively. The experimental soil was sandy clay loam in texture (57.07% sand, 22.32% silt, and 20.61% clay) and acidic in reaction (pH 5.8) with 1.85% organic carbon, 1.43 g/cc bulk density, and 17.36 me/100 g soil CEC. Soil nutrient status was 0.33% total N, 21.2 ppm available P, 143 ppm available K, 794 ppm Ca, and 163 ppm Mg. At field capacity, soil water retention was 22.64% (wet basis) and 29.27% (dry basis).

### 2.2. Plant Material

An aerobic rice line AERON 1, sourced from International Rice Research Institute (IRRI), was used as the plant material in this study. The rice line AERON 1 was selected as the plant material because it performed well under aerobic soil conditions in previous study [21].

### 2.3. Experimental Treatments and Design

 The experimental design was a randomized complete block with three replications. To determine critical period of weed control, a quantitative series of treatments comprising two components, (a) increasing duration of weed interference and (b) increasing length of weed-free period, were imposed. Timing of weed removal was based on the number of weeks after rice seeding. To determine the beginning of the CPWC, the first component, increasing duration of weed interference, was established by allowing the weeds to compete with the crops for 2, 4, 6, and 8 weeks after seeding (WAS) (referred to as weedy plots), after which plots were maintained weed-free until harvest. To evaluate the end of the critical period of the CPWC, the second component, increasing length of weed-free period, was established by maintaining weed-free condition for 2, 4, 6, and 8 WAS (referred to as weed-free plots) before allowing subsequent emerging weeds to compete for the remainder of the growing season. In addition, season long weedy check and weed-free check were included as control. No herbicide was used as weed control was accomplished by hand weeding. The experiment was conducted under naturally occurring population of mixed weed species.

### 2.4. Crop Husbandry

The experimental field was dry-ploughed and harrowed but not puddled during land preparation. Each plot was 5.0 m long and 3.0 m wide and accommodated 12 rows with 25 cm interrow spacing. Rice seeds were directly dry-seeded in rows with 15 cm intrarow spacing at the rate of eight seeds/hill. Each plot was fertilized with triple superphosphate (TSP) and muriate of potash (MP) at 100 kg P/ha and 100 kg K/ha, respectively, as basal application; urea was top-dressed thrice each at 50 kg N/ha at 2, 4, and 6 WAS. Field was maintained under nonsaturated aerobic conditions throughout the growing season. In both the seasons, the trial was primarily rain-fed, but supplemental sprinkler irrigation was applied when needed. Overflow canals were maintained to facilitate drainage whenever heavy rainfall resulted in ponding. Plant protection measures were taken, as needed, to avoid confounding effect of competition with insect and/or disease injury. Different intercultural operations and plant protection measures were conducted following standard practices.

### 2.5. Data Collection

 At each weed removal time, a 25 cm × 25 cm quadrate was randomly placed lengthwise at four spots in each plot for recording weed data. Weeds were clipped to ground level, identified and counted by species, and separately oven dried at 70°C for 72 hours. Weed density (WD) and weed dry weight (WDW) were expressed as no./m^2^ and g/m^2^, respectively. Dominant weed species were identified using the summed dominance ratio (SDR) computed as follows [21]:

(1) $$\;\begin{array}{cc} & \text{SDR}\;\text{of}\;\text{a}\;\text{weed}\;\text{species}=\\ & \;\frac{\left[\text{Relative}\;\text{density}\;\left(\text{RD}\right)+\text{Relative}\;\text{dry}\;\text{weight}\;\left(\text{RDW}\right)\right]}{2}, \end{array}$$

where RD (%) = (Density of a given weed species/Total weed density) × 100, RDW (%) = (Dry weight of a given weed species/Total weed dry weight) × 100.

Four central rows, excluding the harvesting area, were used for data recording. Aboveground crop biomass was recorded at panicle initiation, heading and harvesting stages from ten randomly selected hills of each plot by oven drying at 70°C for 72 hours. At maturity, yield components like panicle/m^2^ and grains/panicle were recorded from ten randomly selected hills. Central 3 m^2^ area of each plot was hand harvested to record grain yield and thousand-seed weight. Grain yield and thousand-seed weight were adjusted to 14% moisture content. Growing degree day (GDD) was accumulated from the date of seeding considering base temperature as 10°C [24]. The time of crop emergence was used as the reference point for the accumulation of GDD. The GDD was calculated as follows:

(2) $$\text{GDD}=\frac{\sum \left({\text{T}}_{\max}+{\text{T}}_{\min}\right)}{2}-{\text{T}}_{b},$$

where *T*_max_ and *T*_min_ are daily maximum and minimum air temperature (°C), and *T*_*b*_ is the base or threshold temperature below which physiological activities are inhibited.

### 2.6. Statistical Analysis

Statistical Analysis System (SAS 9.1) software was used to analyze the data, including analysis of variance (ANOVA) and comparison of means based on a protected LSD procedure at 5% level of probability [25]. Data for both the seasons were analyzed separately. For each season, mean yield across the three blocks was calculated for each treatment and converted to percentage values (relative yield, RY) of the season long weed-free control in each treatment group. To calculate CPWC, RY data for the weedy or weed-free treatments were regressed against the increasing duration of weed interference or increasing length of the weed-free period. A four-parameter logistic equation, proposed by Hall et al. [7] and modified by Knezevic et al. [3], was used to describe the effect of increasing duration of weed interference on relative yield of rice. The Gompertz model [3, 7] was used to provide a good fit to RY as it is influenced by increasing length of the weed-free period. The logistic equation was used to determine the beginning of the CPWC, and the Gompertz equation was used to determine the end of the CPWC for yield loss levels of 5 and 10% chosen arbitrarily [7, 26].

## 3. Results

### 3.1. Weed Composition

 In the experiment, the naturally occurring weed community had a wide weed spectrum including broadlaf, sedges, and grasses. Weed population was mostly dominated by broadleaf weeds with very little contribution of grasses. The weed community composed of 23 species in off-season 2010 and 18 species in main season 2010/2011 representing 12 different families (Table 1). *Physalis heterophylla* Nees, *Scoparia dulcis* L., *Cleome rutidosperma* DC, and *Cyperus rotundus* L. were the most common species dominating the community in both the seasons. *Physalis heterophylla* Nees appeared as the most abundant species in both the seasons with about 58 and 71 plants/m^2^ in off and main seasons, respectively. *Cyperus sphacelatus* Rottb., *Cyperus aromaticus, Axonopus compressus* (Sw.) Beauv, *Amaranthus viridis* L., and *Eclipta prostrata *(L.) L. were present in off-season but not found in main season.

### 3.2. Weed Species Dominance Pattern

 Weed species dominance pattern was not found similar throughout the growing period (Table 2). In both the seasons, *Cyperus rotundus* L., representing sedge group, was the most dominant species at early growth stage of rice, But with the advancement of time, sedges were gradually replaced by broadleaf weeds. Among the grasses, only *Axonopus compressus* (Sw.) Beauv was recorded as one of the five most dominant species at early growth stages in both seasons and thereafter disappeared, whereas *Eleusine indica* (L.) Gaertn. and *Leptochloa chinensis* (L.) Nees were amongst the most dominant species in off-season and main season, respectively, in later growth stages.* Physalis heterophylla* Nees, *Scoparia dulcis* L., and *Cleome rutidosperma* DC were the broadleaf weeds started dominating the community from midgrowth stage of rice till maturity in both the seasons.

### 3.3. Weed Density and Dry Weight

 Weed density and dry weight were recorded at the end of different weed competition periods. Due to aerobic soil conditions, weed density and dry matter were found very high (1241–1311 g/m^2^ and 520–540 g/m^2^, resp.) in both the seasons (Table 3). Weed pressure in terms of weed dry weight was higher in main season than in off-season. In weedy check, weed density and dry weight in main season were recorded as 520/m^2^ and 431 g/m^2^, respectively, while those in off-season were recorded as 471/m^2^ and 390 g/m^2^, respectively. Weed density and dry weight increased with the increasing duration of weed interference period up to 6 WAS and thereafter declined in both the seasons. In contrast, weed density and dry weight decreased with increasing duration of weed-free period.

### 3.4. Rice Biomass

Aboveground crop biomass accumulation by rice variety AERON 1 was significantly influenced by weed interference period at all harvests in both off and main seasons (Table 4). AERON 1 accumulated more biomass in main season than in off-season. Increasing length of weed interference period caused lower biomass accumulation at all the growth stages except at panicle initiation stage, whereas weedy conditions for more than 2 WAS had no significance on biomass production in both season. Adverse effect of increasing weedy period on biomass production increased gradually with the advancement of growth stages. Season long weed competition encountered 63, 73, and 75% penalty in biomass accumulation at panicle initiation, heading, and harvesting stages, respectively, as compared with weed-free check in off-season. While in main season, biomass production was reduced by 62, 74, and 81% at panicle initiation, heading, and harvesting stages, respectively, as a consequence of season long weed interference. Weeding after 8 WAS resulted in no significant increase in biomass accumulation of AERON 1.

### 3.5. Yield Components and Yield

Yield components and grain yield of AERON 1 were significantly influenced by weed competition period in both off and main seasons (Table 5). Number of panicles/m^2^, number of grains/panicle, and thousand-seed weight were increased with the increasing length of weed-free conditions and decreased with the increasing length of weedy conditions. In general, maintaining a weed-free condition beyond 8 WAS did not bring any improvement in the yield components like number of panicles/m^2^ and thousand-seed weight. While, regarding number of grains/panicle, weed interference after 6 WAS had no adverse effect. Season long weed competition resulted in 47, 32, and 13% reduction in number of panicles/m^2^, number of grains/panicle, and thousand-seed weight, respectively, as compared with season long weed-free conditions in off-season, while in main season, the respective values were 56, 27, and 13%. Grain yield of AERON 1 was significantly influenced by weed interference period in both the seasons; grain yield was increased with the increasing length of weed-free period up to 6 WAS after which no significant improvement was observed. In contrast, grain yield was significantly decreased with the increasing span of weed interference period up to 8 WAS and thereafter remained unchanged. Apparently, grain yield was recorded slightly higher in main season than in off-season. Season long weed-free conditions produced a yield advantage of 115 and 122% over season long weedy conditions in off and main seasons, respectively.

### 3.6. Critical Period of Weed Control

**Figure 1:**
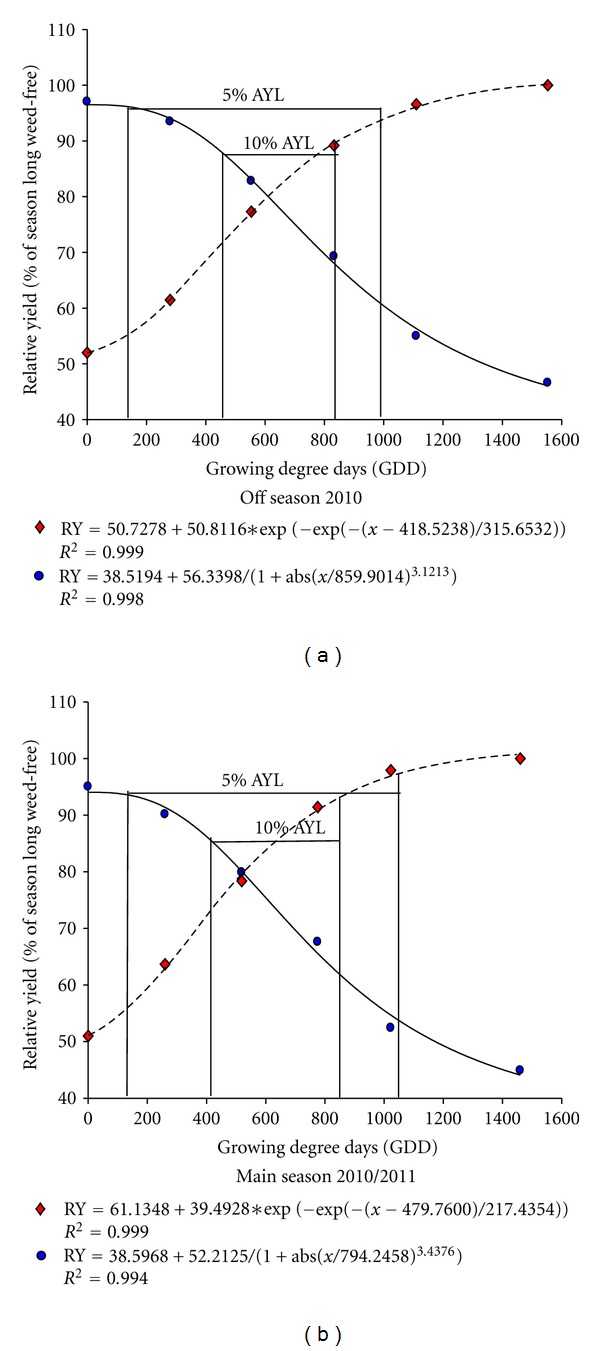
Influence of weed interference on relative yield of aerobic rice variety AERON 1 in the off-season of 2010 and main season of 2010/2011. Increasing duration of weed interference (●) data fitted to the logistic equation; increasing weed-free period (♦) data fitted to the Gompertz equation. The dots and the lines represent observed relative yield and fitted models, respectively. AYL = accepted yield loss; RY = relative yield.

Critical period of weed control (CPWC) was determined by using relative rice yield (% of season long weed-free yield) and growing degree days (GDDs) as quantitative variables in the regression analysis. Rice seeding date was used as the reference point for accumulation of GDD for accounting the possibility of weeds emerging before the rice. The CPWC was determined based on arbitrarily chosen yield loss levels (AYLs) of 5% and 10%, which are judged to be acceptable considering the present economics of weed control. Predicted and observed relative rice yield as affected by weed interference and weed-free periods in off and main seasons are shown in Figure 1. Responses were highly significant as indicated by high *R* ^2^ values. In off-season, the beginning of CPWC based on 10% AYL occurred by 456 GDD corresponding to 23 days after seeding (DAS) (Table 6). In contrast, in main season at the same AYL, weeds required to be removed at 412 GDD, corresponding to 21 DAS. The end of the CPWC at 10% AYL occurred by 832 GDD or 40 DAS in the off-season and 847 GDD or 43 DAS in the main season. At 5% AYL, the onset of CPWC occurred at 137 GDD, relating to 7 DAS in off-season and 131 GDD or 7 DAS in main season. Weeds had to be controlled until 987 GDD, corresponding to 49 DAS in off-season at 5% AYL. In contrast for the main season and the same AYL, rice field should be kept weed-free until 1044 GDD, relating to 53 DAS. It is evident from our study that CPWC of AERON 1 was variable in length between seasons and was a bit longer in main season than in off-season. The beginning of the critical period was relatively stable between seasons; the end, on the other hand, was more variable. The onset of CPWC became delayed and ended earlier as the predetermined AYL increased from 5% to 10%.

## 4. Discussion

Over the past few decades, weed management has been mostly herbicide dependent resulting raised public concern about the residual toxicity of herbicides, which necessitates the development of a less herbicide-dependent weed management system [27]. Study of critical period of weed competition (CPWC) is very crucial for sustainability view point since it optimizes time for implementing and maintaining weed control measures (e.g., timing of herbicide application) and thus reduces ecological risk and improves economics of herbicide application. Therefore, sustainable weed management in aerobic rice relies largely on the identification of CPWC.

The experiment was accomplished under naturally occurring weed population comprising 23 species in off-season and 18 species in main season. Based on summed dominance ratio (SDR), averaged over seasons, the most dominant weed species could be arranged in the order of *Physalis heterophylla > Scoparia dulcis > Cleome rutidosperma > Cyperus rotundus > Fimbristylis miliacea > Eleusine indica > Leptochloa chinensis. *Similarity in weed composition between seasons might be due to proximity of the experimental sites and similarity of cropping pattern and weed management practices. The weed community was mostly dominated by broadleaf weeds followed by sedges and grasses, which is rather different from that of a typical aerobic rice field. In Karnataka, India, Gowda et al. [28] reported grasses and sedges as the predominant weed groups in aerobic rice field. Jaya Suria et al. [29] also accounted from their study with aerobic rice conducted at Penang, Malaysia that grassy weeds constituted about 80% of total weed community. The differences in the floristic composition and dominance pattern of weeds reported in different studies might be due to the variation in the agroecological conditions, cropping pattern, management practices, and weed seed bank composition among the experimental sites [30]. Weed species dominance pattern varied between seasons mostly due to the differences in soil moisture regimes; in main season, rice crop received a total rainfall of 228 mL throughout life cycle, while in off-season, the same received only 180 mL rainfall. Juraimi et al. [18] also reported that rice weed community is strongly influenced by soil moisture conditions. It was evident that broadleaf weeds are found to be more aggressive in main season (82% SDR) than in off-season (54% SDR), which might be due to higher moisture regimes in main season that favored broadleaf weeds more than the grasses or sedges. Abundance of broadleaf weeds under saturated conditions (higher-moisture regimes) has also been reported by Juraimi et al. [31]. Conflicting findings have been reported by Bhagat et al. [32], who recorded dominance of grasses under higher-moisture regimes.

Weed density and dry matter were recorded very high in both the seasons. The weed pressure was higher in main season than in off-season because of more favorable conditions in terms of soil moisture status. Differences in weed dry matter between rice seasons has also been reported by Johnson et al. [15] and Chauhan and Johnson [19]. The high weed pressure as observed in this study confirms the findings of many other researchers [29, 33, 34], who reported that weed pressure in aerobic rice is the highest among the rice ecosystems. In season long weedy check, weed dry matter in the previous study [19] with aerobic rice ranged from 458 to 692 g/m^2^ in between seasons, whereas, in this study, weed dry matter ranged from 390 to 431 g/m^2^, a 14–37% lower weed dry matter. The dissimilarity could be due to the contrasting rice variety, weed flora, soil moisture regimes, and agroclimatic conditions between experimental sites.

Biomass accumulation by AERON 1 was adversely affected by the increasing length of weed interference period and, conversely, favorably influenced by the increasing span of weed-free period up to 6 or 8 WAS. Maintaining weed-free conditions after 6 or 8 WAS failed to improve biomass accumulation of rice. Rice grown in a competitive advantage in comparison with weed will grow better than its counterpart and vice versa. At early crop growth stage, weeds may be better competitor than the crop, which is likely due to competitive advantages for the weeds in terms of preemption of resources. But with the advancement of times, crop start dominating over weeds, and after a certain stage, weed is no more a threat for crop growth, controlling weeds after that resulting no significant enhancement in crop growth. Similar findings have been reported by many researchers [17, 18, 35], who observed that weed interference up to a certain growth stage had negative impact on rice growth.

Rice grain yield decreased with prolonged delays in weed removal; conversely, grain yield increased with the increasing length of weed-free period in both the seasons. Weed competition throughout reduced crop yield by approximate 55% in both seasons as compared with season long weed-free period. These values are very close to those reported in a previous study, where season long weed competition reduced yield by approximately 50% [15]. Juraimi et al. [18] recorded 79 and 66% yield reduction in rice due to weed competition till harvest in flooded and saturated conditions, respectively. Chauhan and Johnson [19], on the contrary, reported as high as 95% yield reduction in aerobic rice due to weed competition throughout the crop growing season. These contrasting findings might be due to differences in rice variety, agroclimatic conditions, soil moisture regimes, and weed flora among the experimental sites. Prolonged weed competition resulted in reduced biomass accumulation and lesser panicles/m^2^, grains/panicle, and thousand-seed weight which ultimately translated into lower grain yield. Increased biomass accumulation by weeds with the increasing span of weed interference period might also be a plausible cause of yield reduction in rice. As Woolley et al. [36] stated, weed dry matter has been found to be highly correlated with crop yield loss.

Based on 5% AYL, our results imply that under similar experimental conditions rice can tolerate weed interference until 137 GDD or 7 DAS in off-season and 131 GDD or 7 DAS in main season, suggesting that control measures should start at that stage; the crop should be kept weed-free until 987 GDD corresponding to 49 DAS in off-season and 1044 GDD corresponding to 53 DAS in main season in order to prevent appreciable economic yield loss. Therefore, weed control measures must begin as soon as possible after rice emergence to prevent yield loss more than 5% in both seasons. At 10% AYL, the beginning of the CPWC was determined 456 GDD for off-season and 412 GDD in main season; to prevent more than acceptable yield loss, aerobic rice field should be maintained weed-free until 832 GDDs have been accumulated in off-season, while in main season, fields should continue to be scouted until 847 GDDs have been accumulated. This equates to controlling weeds from 23 to 40 DAS and 21 to 43 DAS in off and main seasons, respectively. This implies that keeping rice weed-free for that stipulated period is equivalent to keeping rice weed-free season long; presence of weeds before or after that period will not pose a threat [5], and hence, there may be very little benefit of subsequent weed control.

As Zimdahl [12] stated, the CPWC is “not an inherent property of the crop,” and CPWC of a particular crop is weed species, site, and season specific [13]. Johnson et al. [15] estimated CPWC for lowland irrigated rice as 0–32 DAS in wet season and 4–83 DAS in dry season to obtain 95% yield in West Africa. In Malaysia, based on the 5% yield loss, Begum et al. [17] concluded that flood-irrigated rice must be kept free from weed competition from 14 to 28 DAS, while Juraimi et al. [37] suggested that direct-seeded rice should be kept weed-free for 2–71 DAS in saturated condition and 15–73 DAS in flooded condition. In the Philippines, Chauhan and Johnson [19] estimated CPWC of aerobic rice as between 18 and 52 DAS to obtain 95% of weed-free yield. Thus, CPWC is highly variable and is largely dependent on the relationship of crop seeding date to the emergence periodicity for the weed community of a particular site [26].

The onset of the critical period was found relatively stable between seasons, while the end was more variable. This phenomenon is supported by many researchers [4, 38, 39], who opined that the end of CPWC was variable and highly dependent on density, competitiveness, and emergence periodicity of the weed population. The CPWC in main season started earlier and lasted longer, as compared to off-season. A long critical period is the indication of less competitive crop or more competitive weeds and vice versa [1, 6]. A possible reason for starting earlier and lasting longer of CPWC in main season might be the conditions favorable for weed germination and growth. Main season received more rainfall than off-season which might provide weeds an advantage over the rice crop. Juraimi et al. [18] also estimated longer CPWC of rice in main season and shorter one in off-season in both flooded and saturated conditions. Johnson et al. [15], on the contrary, observed differences in CPWC between seasons in irrigated lowland rice.

The study portrays the significance of CPWC determination for sustainable weed management in aerobic rice. The practical implication of this study is that under the similar experimental conditions aerobic rice field should be kept weed-free during 7–49 DAS in off-season and 7–53 DAS in main season to achieve 95% of weed-free yield, and 23–40 DAS in off-season and 21–43 DAS in main season to achieve 90% weed-free yield. Since 5% yield loss level would not be practical from economic view point, a 10% yield loss may be considered excellent in terms of economic return, and this level can be achieved by early postemergence application of herbicide or weeding between 10 and 15 DAS followed by a post emergence application or weeding between 30 and 35 DAS. Since weeds emerge after this period are supposed to cause no substantial yield looses, the need for applying additional herbicides or weeding more than 2 times as practiced by most farmers would not be warranted and could lead to significant cost savings. Nevertheless, weed management can be extended beyond that period if the objective is not only to have higher yield but also to avoid weed seed rain to prevent buildup of the weed seed bank, which is of major concern for long-term sustainability of weed management.

## Figures and Tables

**Table 1:** Weed composition in season long weedy plots of aerobic rice (Off-season 2010 and main season 2010/2011; summed dominance ratio (SDR ± SE)).

Scientific name	Family name	Weed type	Summed dominance ratio	
			Off-season 2010	Main season 2010/2011
*Alternanthera sessilis *(L.) R. Br. Ex DC.	Amaranthaceae	B	0.8 ± 0.19	0.6 ± 0.11
*Amaranthus viridis* L.	Amaranthaceae	B	0.8 ± 0.25	—
*Axonopus compressus* (Sw.) Beauv	Poaceae	G	1.1 ± 0.51	—
*Cleome rutidosperma* DC	Capparidaceae	B	10.3 ± 2.6	15.3 ± 5.1
*Cyperus aromaticus*	Cyperaceae	S	2.5 ± 0.81	—
*Cyperus distans*	Cyperaceae	S	4.1 ± 1.2	2.9 ± 0.86
*Cyperus rotundus* L.	Cyperaceae	S	9.2 ± 1.9	7.2 ± 2.8
*Cyperus sphacelatus* Rottb.	Cyperaceae	S	3.5 ± 0.98	—
*Digitaria ciliaris *(Retz.) Koel	Poaceae	G	1.5 ± 0.51	0.3 ± 0.11
*Echinochloa colona *(L.) Link	Poaceae	G	3.0 ± 0.76	2.2 ± 1.1
*Eclipta prostrata *(L.) L.	Asteraceae	B	0.3 ± 0.12	—
*Eleusine indica* (L.) Gaertn.	Poaceae	G	7.6 ± 2.2	1.7 ± 0.56
*Emilia sonchifolia *(L.) DC. Ex Wight	Asteraceae	B	0.6 ± 0.10	0.4 ± 0.10
*Euphorbia hirta* L.	Euphorbiaceae	B	0.7 ± 0.17	4.3 ± 1.34
*Fimbristylis miliacea* (L.) Vahl	Cyperaceae	S	6.4 ± 2.0	3.2 ± 1.2
*Hedyotis corymbosa *(L.) Lam.	Rubiaceae	B	1.4 ± 0.39	3.7 ± 1.2
*Hyptis brevipes *Poit	Lamiaceae	B	1.0 ± 0.22	0.7 ± 0.23
*Jussiaea linifolia *Vahl	Onagraceae	B	2.2 ± 0.25	6.6 ± 2.9
*Leptochloa chinensis* (L.) Nees	Poaceae	G	7.0 ± 1.3	1.1 ± 0.30
*Mimosa pudica* L.	Fabaceae	B	1.3 ± 0.36	6.4 ± 1.3
*Phyllanthus niruri *L.	Euphorbiaceae	B	1.9 ± 0.42	5.1 ± 0.84
*Physalis heterophylla* Nees	Solanaceae	B	20.8 ± 5.8	23.7 ± 7.2
*Scoparia dulcis* L.	Scrophulariaceae	B	12.0 ± 3.4	14.6 ± 4.6

B = broadleaf; S = sedge; G = grass.

**Table 2:** Five most dominant weed species at the end of different weedy periods in the two seasons (off-season 2010 and main season 2010/2011, with summed dominance ratios (SDRs) followed by standard error (SE)).

Weed species	Weedy 2 weeks	Weedy 4 weeks	Weedy 6 weeks	Weedy 8 weeks	Season long weedy
	Off-season 2010				
*Axonopus compressus*	13.7 ± 3.7	—	—	—	—
*Cleome rutidosperma* DC	—	11.4 ± 5.4	13.5 ± 3.2	15.7 ± 2.8	10.3 ± 2.6
*Cyperus distans*	15.5 ± 4.4	—	—	—	—
*Cyperus rotundus* L.	39.9 ± 16.2	31.4 ± 8.3	11.2 ± 4.0	9.9 ± 2.9	9.2 ± 1.9
*Eleusine indica* (L.) Gaertn.	—	—	—	—	7.6 ± 2.2
*Fimbristylis miliacea* (L.) Vahl	—	7.2 ± 1.8	6.1 ± 2.5	6.7 ± 3.4	—
*Physalis heterophylla* Nees	9.2 ± 2.4	17.6 ± 3.9	28.2 ± 7.5	26.5 ± 4.6	20.8 ± 5.8
*Scoparia dulcis* L.	7.1 ± 3.1	15.8 ± 5.1	15.9 ± 6.8	18.0 ± 5.2	12.0 ± 3.4

	Main season 2010/2011				
*Axonopus compressus*	5.7 ± 0.9	—	—	—	
*Cleome rutidosperma* DC	16.4 ± 7.3	18.7 ± 5.7	20.1 ± 6.7	17.6 ± 7.9	15.3 ± 5.1
*Cyperus rotundus* L.	33.6 ± 8.6	26.2 ± 8.2	13.2 ± 2.3	8.3 ± 3.5	7.2 ± 2.8
*Jussiaea linifolia *Vahl	—	—	—	—	6.6 ± 2.9
*Leptochloa chinensis* (L.) Nees	—	—	3.6 ± 0.7	7.8 ± 2.7	
*Mimosa pudica* L.	—	6.6 ± 2.5	—	—	
*Physalis heterophylla* Nees	22.3 ± 3.8	27.4 ± 5.9	29.5 ± 13.1	26.5 ± 6.8	23.7 ± 7.2
*Scoparia dulcis* L.	2.5 ± 0.4	6.3 ± 1.4	11.7 ± 3.5	10.4 ± 5.2	14.6 ± 4.6

**Table 3:** Effect of duration of weed competition on density and dry weight of weeds in both seasons (off-season 2010 and main season 2010/2011).

Weed competition period	Off-season 2010		Main season 2010/2011	
	Density (no./m^2^)	Dry matter (g/m^2^)	Density (no./m^2^)	Dry matter (g/m^2^)
Weedy until 2 WAS	879.67c	155.33de	950.33c	188.33e
Weedy until 4 WAS	1073.33b	215.00cd	1128.00b	239.0de
Weedy until 6 WAS	1241.00a	520.33a	1311.00a	548.33a
Weedy until 8 WAS	979.67b	503.00a	1033.33bc	532.33a
Weedy check	471.00d	390.00b	520.67d	431.67b
Weed-free until 2 WAS	473.00d	342.00b	506.00d	364.67c
Weed-free until 4 WAS	380.67d	256.67c	421.33d	286.0d
Weed-free until 6 WAS	206.00e	164.33de	224.33e	181.67e
Weed-free until 8 WAS	102.33f	107.33e	123.67e	101.33f

Data for weedy treatments were taken at the time of weed removal, whereas data for weed-free treatments were taken at the time of rice harvest.

Within a column for each factor, means sharing same alphabets are not significantly different at *P* = 0.05 probability level according to least significant difference test.

WAS = weeks after seeding.

**Table 4:** Effect of weed competition period on aboveground crop biomass production in aerobic rice variety AERON 1 at different growth stages in both seasons (off-season 2010 and main season 2010/2011 (g/m^2^)).

Weed competition period	Off-season 2010			Main season 2010/2011		
	Panicle initiation stage	Heading stage	Harvest	Panicle initiation stage	Heading stage	Harvest
Weedy until 2 WAS	191.77a	364.35b	660.71ab	201.89a	328.23ab	669.60b
Weedy until 4 WAS	83.20c	293.42c	631.00b	81.40c	306.60b	652.00b
Weedy until 6 WAS	80.32c	215.00d	443.53d	76.00c	241.14c	431.30d
Weedy until 8 WAS	77.43c	119.42f	235.42e	75.28c	111.00e	205.55e
Weedy check	77.59c	108.29f	169.00f	75.67c	91.59e	130.49f
Weed-free until 2 WAS	165.30b	158.00e	247.48e	151.35b	169.47d	253.67e
Weed-free until 4 WAS	208.57a	302.66c	553.00c	185.48a	302.62b	589.07c
Weed-free until 6 WAS	208.98a	364.7b	664.36ab	197.73a	336.67ab	654.23b
Weed-free until 8 WAS	210.10a	381.85ab	673.00ab	204.16a	353.72a	687.58ab
Weed-free check	209.00a	406.00a	679.45a	202.31a	359.87a	729.33a

Within a column for each factor, means sharing same alphabets are not significantly different at *P* = 0.05 probability level according to least significant difference test.

WAS = weeks after seeding.

**Table 5:** Effect of weed competition period on yield components of aerobic rice variety AERON 1 in both seasons (off-season 2010 and main season 2010/2011).

Weed competition period	Off-season 2010				Main season 2010/2011			
	Panicles (no./m^2^)	Grains/panicle (no.)	1000-seed weight (g)	Grain yield (t/ha)	Panicles (no./m^2^)	Grains/panicle (no.)	1000-seed weight (g)	Grain yield (t/ha)
Weedy until 2 WAS	191.0ab	72.6ab	27.2ab	3.37ab	177.0a–c	72.3ab	27.5ab	3.46ab
Weedy until 4 WAS	163.6bc	68.3a–c	27.0b	2.99a–c	162.3bc	65.6a–c	27.0cd	3.07bc
Weedy until 6 WAS	150.3cd	59.0b–d	26.5bc	2.5c–e	148.0c	62.6a–c	26.2f	2.59d
Weedy until 8 WAS	134.0c–e	51.0d	26.1cd	1.98e–f	147.3cd	60.3bc	25.0g	2.01e
Weedy check	104.0e	50.3d	24.2e	1.68f	91.6e	53.3c	24.2h	1.73e
Weed-free until 2 WAS	118.3de	55.0cd	25.5d	2.22df	110.3de	59.6bc	26.4ef	2.43d
Weed-free until 4 WAS	135.3c–e	55.3cd	26.1cd	2.79b–d	140.6cd	61.6bc	26.6e	3.01c
Weed-free until 6 WAS	152.6cd	67.3a–c	26.5bc	3.22ab	165.3bc	63.6a–c	26.7de	3.51a
Weed-free until 8 WAS	193.3ab	72.3ab	27.1ab	3.49a	198.3a	67.3ab	27.1bc	3.76a
Weed-free check	199.0a	74.3a	27.8a	3.61a	205.6a	73.3a	27.8a	3.84a

Within a column for each factor, means sharing same alphabets are not significantly different at *P* = 0.05 probability level according to least significant difference test.

WAS = weeks after seeding.

**Table 6:** Estimated critical periods of weed control for two acceptable levels of crop losses in both seasons (off-season 2010 and main season 2010/2011).

Yield loss levels (%)	Critical period							
	Off-season 2010				Main season 2010/2011			
	Onset		End		Onset		End	
	Growing degree days	Days after seeding	Growing degree days	Days after seeding	Growing degree days	Days after seeding	Growing degree days	Days after seeding
5	137	7	987	49	131	7	1044	53
10	456	23	832	40	412	21	847	43
